# Investigations of the ADOR Process Using Solid-State NMR Spectroscopy

**DOI:** 10.1021/acs.cgd.3c01037

**Published:** 2023-11-15

**Authors:** Cameron M. Rice, Olivia J. Dovernor, Russell E. Morris, Sharon E. Ashbrook

**Affiliations:** School of Chemistry, EaStCHEM and Centre of Magnetic Resonance, University of St Andrews, Purdie Building, St Andrews KY16 9ST, U.K.

## Abstract

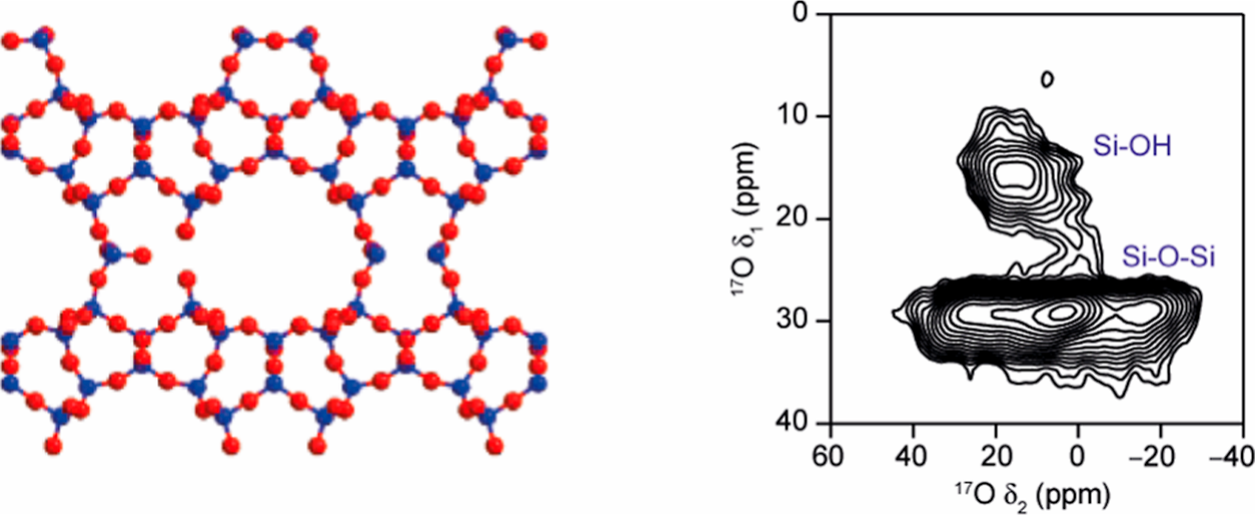

The assembly–disassembly–organization–reassembly (ADOR) process for the transformation of zeolite **UTL** using water has been studied by using ^29^Si and ^17^O solid-state NMR spectroscopy. The results show that the intermediate materials that are formed during the reaction are extremely dynamic and that the process involves both irreversible changes in structure that define the overall pathway and reversible lability of the Si–O–Si linkages that have no effect on the overall structure. The combination of processes occurring during the ADOR reaction means that the mechanism is considerably more complex than initially proposed.

## Abstract 2

Solid-state NMR has been used to provide new information on the mechanism of the synthesis of a zeolite using the ADOR process. The results indicate a more complex mechanism than was previously described, developing the information on the changes of local structure that occur during the process.

## 1 Introduction

**Figure 1:**
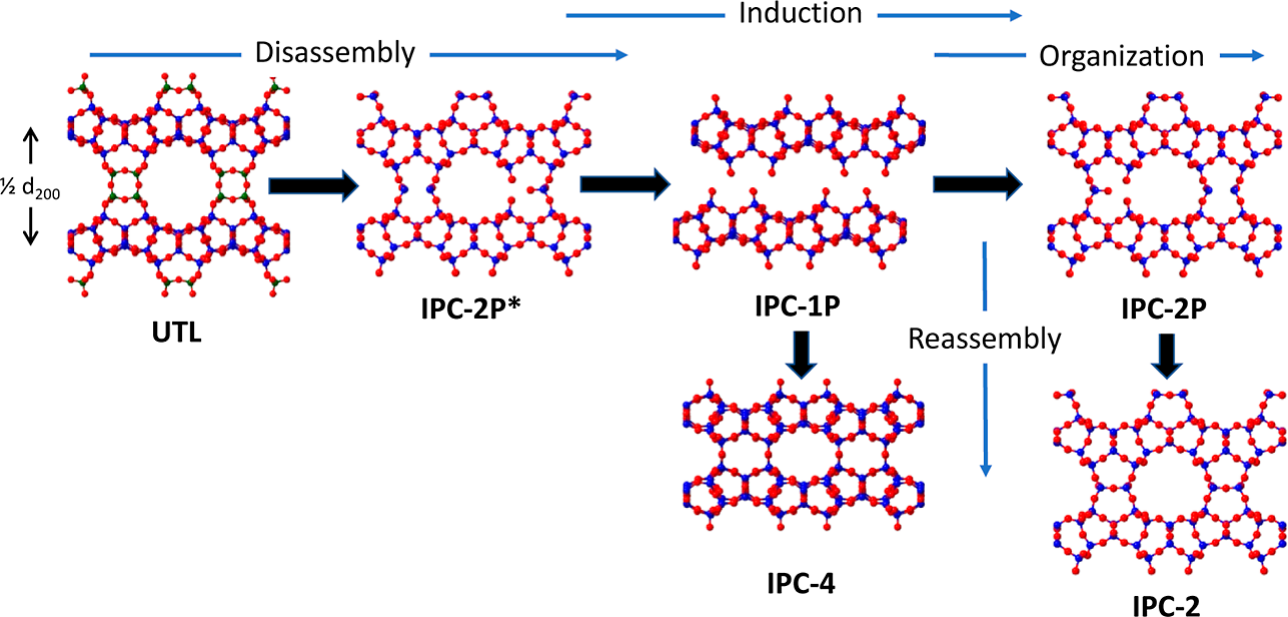
Schematic of the ADOR process showing the different intermediates discussed. (Note that IPC-6 is not shown but is an intermediate material between IPC-1P and IPC-2P). Marked on the diagram is the relationship between the *d*_200_ spacing and interlayer spacing in the materials. Note that the Assembly step is not shown, as it is the initial formation of the parent zeolite, **UTL**.

Zeolites remain one of the most important classes of porous solid, with extensive applications across a wide range of industries.^1^ The assembly–disassembly–organization–reassembly (ADOR) process is a recently established method for synthesizing new high-silica zeolite materials from pre-existing zeolites by exploiting inherent weaknesses within their structure.^2^ The process itself consists of the basic steps shown in Figure 1, with the final framework being formed through a reassembly step that involves a condensation reaction—a step that is now recognized as leading to novel materials^3,4^ when compared to traditional hydrothermal synthesis.^5^ To work, the reaction system requires a starting (or parent) zeolite material possessing an instability that can be exploited under mild reaction conditions.^6^ Germanosilicate zeolites provide an ideal system for application of the ADOR protocol because of the hydrolytically sensitive Ge–O that can be selectively targeted over the more stable Si–O bonds.^7^ The ADOR reactivity of germanosilicate zeolites has proven to be very versatile under a variety of conditions: parent material, temperature, pH, solvent system, pressure, reaction volume, time, reaction setup, and atmosphere.^8,9^ These investigations have produced a range of materials with a varied composition, pore structure, and catalytic activity.^10^

To date, six different germanosilicate zeolites have been found to be “ADORable”, producing 13 daughter zeolites with novel topologies. The most successful parent zeolite for producing new materials has been **UTL**(11,12) and using the ADOR protocol has produced seven new daughter materials to date.^13,14^ The structure of **UTL** comprises silicon-rich layers that are linked by cubic germanosilicate units, called double four rings (d4r). On disassembly, these d4r units are removed to form IPC-1P (Figure 1). IPC-1P can either be reassembled directly (by high temperature calcination at >550 °C) to form zeolite IPC-4 or allowed to rearrange further to form new intermediates IPC-6P and IPC-2P. Calcination of these intermediates produces new zeolites that contain Si_4_O_4_ rings (called single four rings, s4r) between half the layers in the case of IPC-6 or all the layers in the case of IPC-2. The reason for the success of **UTL** as a parent zeolite in the ADOR process is the excellent stability of the layers formed on disassembly—unlike layers from other parent zeolites, they are not susceptible to degradation under the conditions used. Scanning electron microscopy studies show that the morphology of the crystallites does not change during the ADOR process,^15^ and the Si/Ge ratio changes from ∼4.5 to >50–100 at early stages during the reaction, indicating that almost all the Ge is lost.^13,14^ Despite this success, the exact process by which the parent **UTL** zeolite disassembles and rearranges to form isolatable crystalline intermediates and zeolite precursors remains unclear.

In addition to the irreversible changes to structure that occur on hydrolytic removal of the germanium from the **UTL**, recent work has also shown that aluminosilicate zeolites on exposure to water, even at room temperature, display lability of the Si–O–Si bonds without an overall change to the structure.^16,17^ This lability is best studied by solid-state NMR spectroscopy as substitution of ^16^O by ^17^O is easily followed in such experiments.^18−20^ So while the initial publications suggested a rather simple stepwise mechanism for the ADOR reaction, in reality, it is a much more complex process involving several different simultaneous changes to the structure. In this paper, we further explore the ADOR process, using solid-state NMR experiments to study both the changes in structure that occur during the disassembly and organization stages of the process, as well as demonstrating that reversible oxygen isotopic exchange is also occurring at significant rates. This confirms that the ADOR process is an even more complex reaction than was first thought.

Contributing factors to our gap in understanding of the ADOR mechanism include the unknown chemical composition of some early intermediates of the process; the speed with which the ADORable species react and rearrange in hot water during hydrolysis (which makes understanding the early stages of the process difficult); the disparity between the long-range order and local structural order of certain intermediates; and the significance of the postdisassembly and prereassembly induction period. Prior to this work, very little was known about the induction period in particular. Here, through powder X-ray diffraction (PXRD), local structural disorder analysis, and the development of novel protocols for ^17^O enrichment, enabling monitoring using solid-state NMR spectroscopy, specific reaction intermediates along the ADOR process have been characterized, giving novel insights to the disassembly and rearrangement of **UTL** in water.

## 2 Results

**Figure 2:**
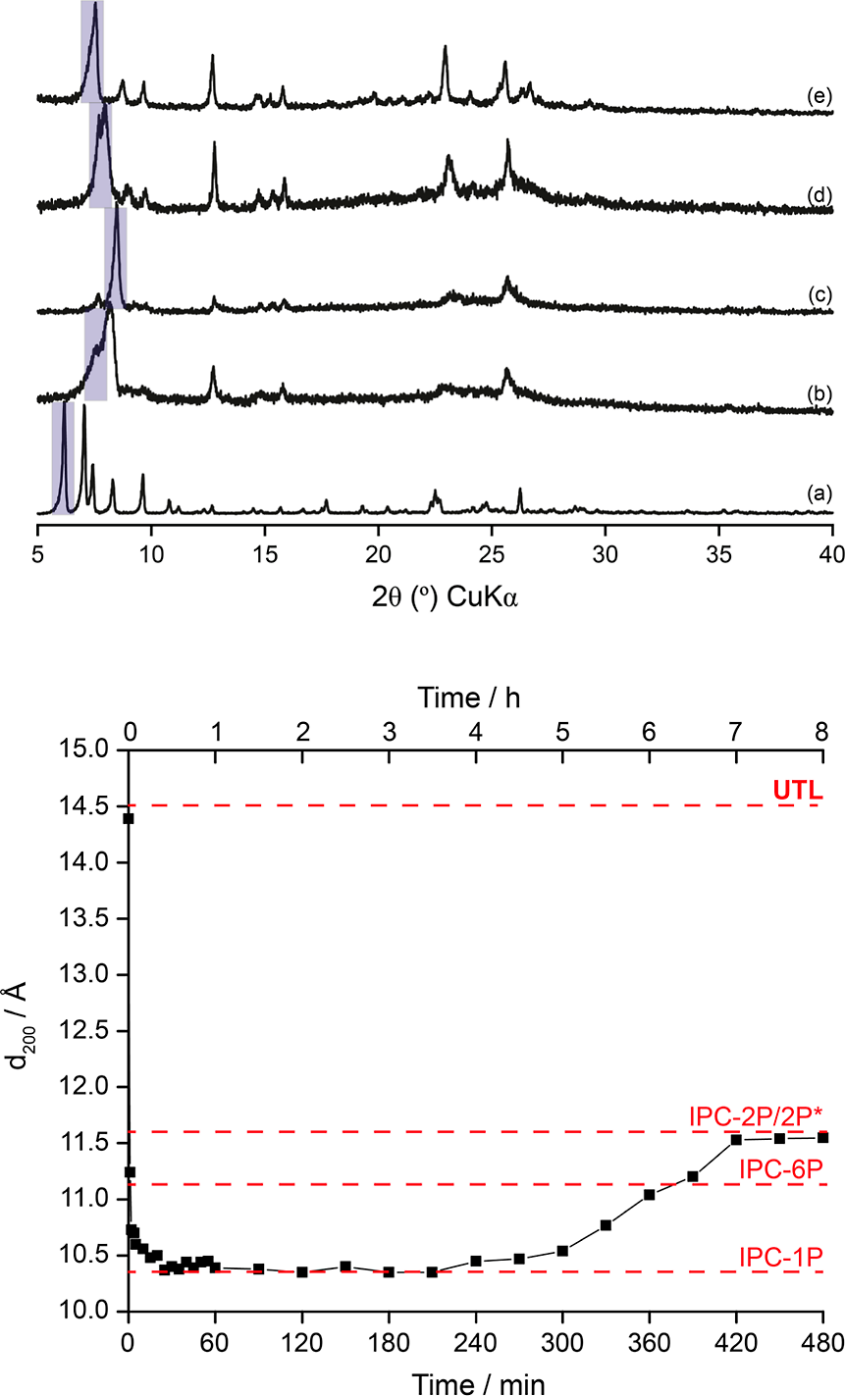
(Top) Plot showing the evolution of the PXRD patterns of the ADOR intermediates after reaction in water at 92 ^*°*^C. (a) Parent Ge-**UTL** Si/Ge = 4.5, (b) IPC-2P*, isolated after 1 min, (c) IPC-1P, isolated after 1 h, (d) IPC-6P, isolated after 6 h, and (e) IPC-2P, isolated after 8 h. The 200 reflection is highlighted in blue for each material. **(Bottom**) The evolution of the *d*_200_ spacing (Å) with time over the course of the ADOR process. The *d*-spacings for each of the intermediate materials are shown by red lines. In this reaction, the induction period lasts from ∼60 to ∼200 min.

Figure 2 shows how PXRD can be used to follow the evolution of the ADOR process. The 200 reflection, present in all diffraction patterns after ADOR transformations of **UTL**, can be used to follow the disassembly and organization steps of the reaction by identifying the intermediates. Development of a standard protocol by Henkelis et al.^21,22^ provided a starting point for optimization of the best method to follow the ADOR transformation in water. Owing to the small sample volume required for PXRD analysis, sampling could take place frequently even at high temperatures: every minute for the first 5 min and thereafter every 5 min up to 60 min, after which every 30 min. This method proved to be effective at capturing each stage of the reaction (Figure 2). At 100 °C, the disassembly step is completed after approximately 5 min, with organization (rearrangement) starting after a 60 min induction period and complete within 4 h of the reaction starting. Thus, a complete ADOR rearrangement can be comfortably captured at 100 °C in 8 h. In this study, however, larger sample sizes applicable for solid-state NMR experiments (using 4 mm rotors for ^29^Si and 4 or 3.2 mm rotors for ^17^O) were required at specified intervals. It was found that it was not possible to achieve this at higher temperatures as the time taken to remove sufficient product from the reaction flask was greater than the evolution of intermediates, particularly within the first 60 min of the reaction. Consequently, the reaction temperature was reduced to 92 °C, as this slowed the rate of disassembly sufficiently without compromising the reaction mechanism.

To ensure consistency in analysis of the ADOR hydrolysis reaction in water for Ge-**UTL,** a set of standard hydrolysis protocols and reactions were devised. To aid this, a full hydrolysis profile for this reaction system was required and a complete PXRD profile of the 92 °C hydrolysis was obtained (Figure 2), using a standard solid/water ratio of 1:200 and a starting **UTL** material of Si/Ge = 4.5. Specific reaction details are found in the Materials and Methods Section.

The reaction follows the pathway shown in Figure 2 at 92 °C in water for Ge-**UTL** hydrolysis, although it was found to be slightly slower than that reported by Henkelis.^20^ Initially, the material rapidly hydrolyzes through IPC-2P* (one min) to IPC-1P (*≈*20 min), before a 3 h induction period, after which rearrangement occurs and the material passes through IPC-6P (6 h), before forming the end product IPC-2P (7 h). The expected (based on ideal materials) and measured 200 reflection positions for key intermediates are listed in Table 1. Note that the high rate of reaction of the initial disassembly step means that it is difficult to isolate IPC-2P* at exactly the same time as that expected, leading to the discrepancy noted in Table 1.

**Table 1:** Key Intermediates in the ADOR Hydrolysis Transformation for Ge-**UTL** in Water at 92 °C and Their *d*_200_ and CuK_α_ 2θ Reflection Positions^a^

intermediate	expected *d*_200_/Å	experimental *d*_200_/Å	experimental Cu K_α_ 2θ (deg)	time formed
**UTL**	14.53	14.39	6.14	n/a
IPC-2P*	11.62	11.21	7.81	1 min
IPC-1P	10.40	10.42	8.48	30 min
IPC-6P	11.15	11.20	7.87	360 min
IPC-2P	11.62	11.73	7.58	420 min

a Note that the initial stages of the reaction are very fast, making it difficult to isolate IPC-2P* with exactly the expected *d*_200_.

### 2.1 Following the ADOR Process Using Ex Situ ^29^Si NMR Spectroscopy

Although PXRD is effective at capturing the formation and lifetimes of different intermediates generated during hydrolysis, it gives an average picture of the long-range order of the system and is unable to offer significant insight into the mechanism of disassembly or organization taking place—this is especially true in the case of the partially disordered intermediates as is the case here. However, the sensitivity to the local structural environment of solid-state NMR spectroscopy and the ability to selectively probe (when desired) certain species present in ADOR intermediates, such as Si–OH, using ^1^H–^29^Si cross-polarization (CP) experiments, make it well-suited to study materials, processes and intermediates generated through the ADOR process.

Solid-state NMR spectroscopy has been used previously to study the ADOR process, shedding light on several important structural and mechanistic aspects unattainable through diffraction-based approaches.^23,24^^29^Si NMR spectroscopy has been used to characterize the chemical environments of new zeolites and hydrolytic intermediates formed through the ADOR process, providing information on defect levels and disparities between expected silanol concentrations from diffraction models and the actual level of disassembly using Q^3^:Q^4^ ratios.^25−27^ This is important because in defect-free, fully connected zeolites, there will be no Q^3^ (HO–**Si** (OSi)_3_) species present, but as disassembly occurs, these species are formed because of removal of framework atoms. Of course, real samples are never perfectly defect free, and even in the parent UTL material, there are some Q^3^ species present, which can be seen at—105 ppm in the spectra of the starting UTL materials. Such resonances account for ∼6% of the ^29^Si NMR signals. However, this is probably a considerable overestimate of the real amount of Q^3^ in UTL, as these resonances do overlap with some of the signals from Si–O–Ge linkages. The number of Q^3^ species in UTL is therefore much lower than that expected in all of the ADOR intermediates. The expected Q^3^:Q^4^ ratio can be calculated for ideal intermediate structures, giving a second method by which intermediates can be identified. Further, the study of degermanation processes in germanosilicate zeolites also uncovered the chemical shifts of silicon atoms located within the d4r of ADORable zeolites—something also achieved through ^11^B NMR and its preferential location within the **UTL** framework.^28,29^ NMR spectroscopy has been employed in the extension of the ADOR process to catalytic applications where ^27^Al NMR studies have monitored both synthetic and postsynthetic incorporation of Al into the **UTL** structure, with time-resolved studies offering insight into rate of incorporation under different reaction conditions.^30,31^^19^F NMR spectroscopy has also proven valuable in the characterization of ADORable germanosilicate materials by identifying germanium populations and locations within the d4r units.^32^ The preference of Ge to organize in 4r sheets within d4r of **UTL**, rather than randomly, is postulated to be a driving factor for the success of **UTL**-derived ADOR processes.^33^ Further, ^17^O NMR spectroscopy experiments have shown a surprising level of exchange of framework oxygen sites for ^17^O isotope in ^17^O-enriched hydrolyses of **UTL** and have demonstrated how the hydrolysis mechanism followed depends on system treatment.^22,34^ Here, ^29^Si NMR spectroscopy is used to explore the changes in the local structure of Si species in the hydrolytic intermediate structures, as they disassemble and organize to crystallographically distinct materials. Changes to the local structure of the IPC-1P intermediate during the induction period are also investigated.

**Figure 3:**
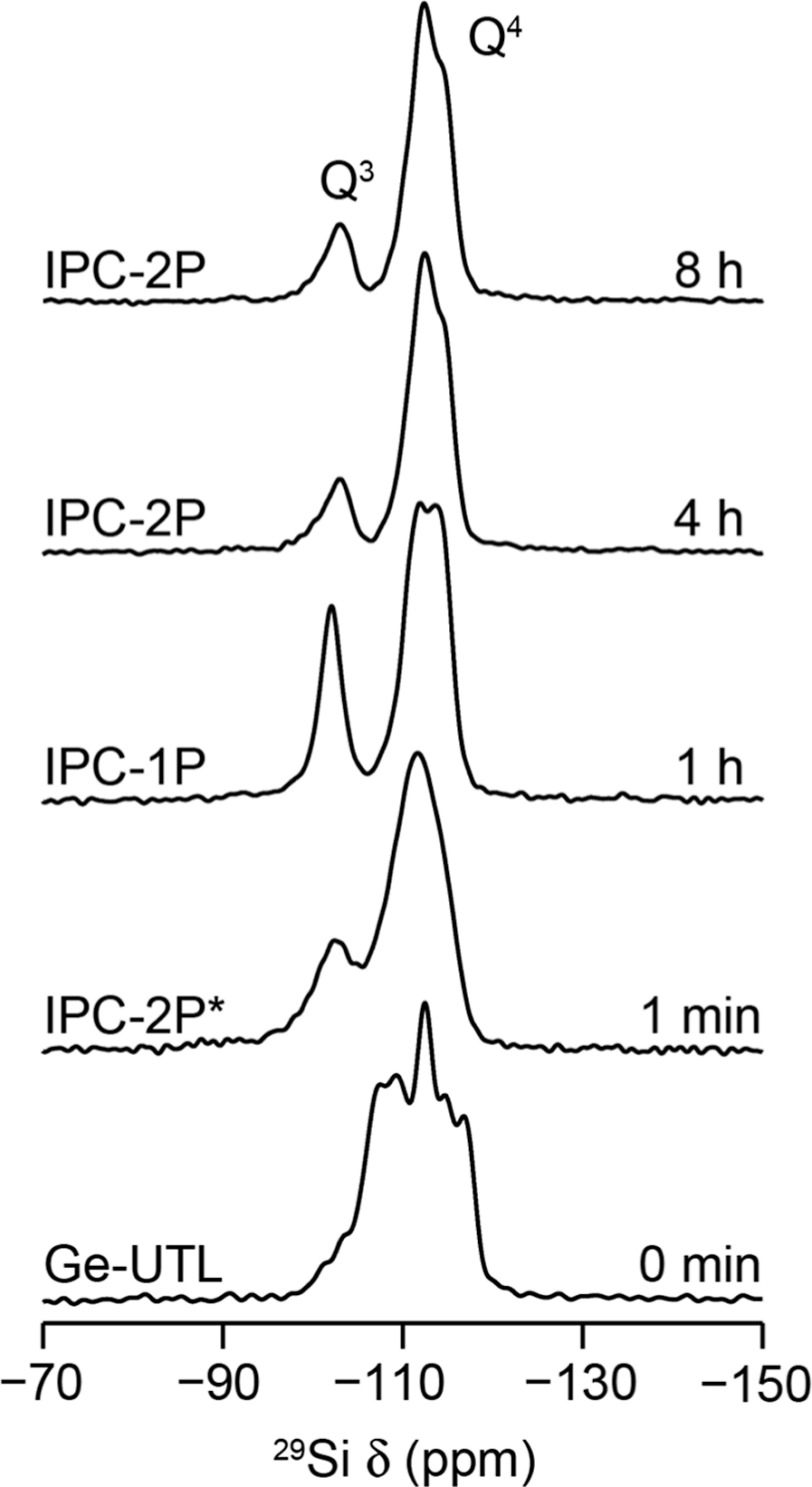
^29^Si (9.4 T, 10 kHz MAS) NMR spectra of products obtained from the hydrolysis of **UTL** in water at 100 °C using hydrolyses for varying amounts of time. PXRD-derived product identification (left) and reaction time (right) are indicated on each spectrum.

To learn more about the changes to the local structure of ADOR intermediates formed during the hydrolysis of **UTL** in water, a series of standard hydrolysis reactions were performed at 100 °C, taking samples after 1, 60, 320, and 480 min. The kinetics of the ADOR hydrolysis reaction at this temperature mean that the expected products at each of these intervals are expected to be IPC-2P*, IPC-1P, IPC-2P, and IPC-2P. Results from diffraction (Table 2) and ^29^Si NMR spectroscopic (Figure 3) analyses of the materials are compared.

**Table 2:** Comparison of the *d*_200_ Spacing (Å) of the Products Obtained by Standard ADOR Hydrolyses of Ge-**UTL** in Water at 100 °C with the Expected Spacings

hydrolysis time/min	expected product	expected *d*_200_/Å	experimental *d*_200_/Å	experimental product
0	**UTL**	14.53	14.39	**UTL**
1	IPC-2P*	11.62	11.85	IPC-2P*
60	IPC-1P	10.40	10.58	IPC-1P
240	IPC-2P	11.62	11.58	IPC-2P
480	IPC-2P	11.62	11.60	IPC-2P

**Figure 4:**
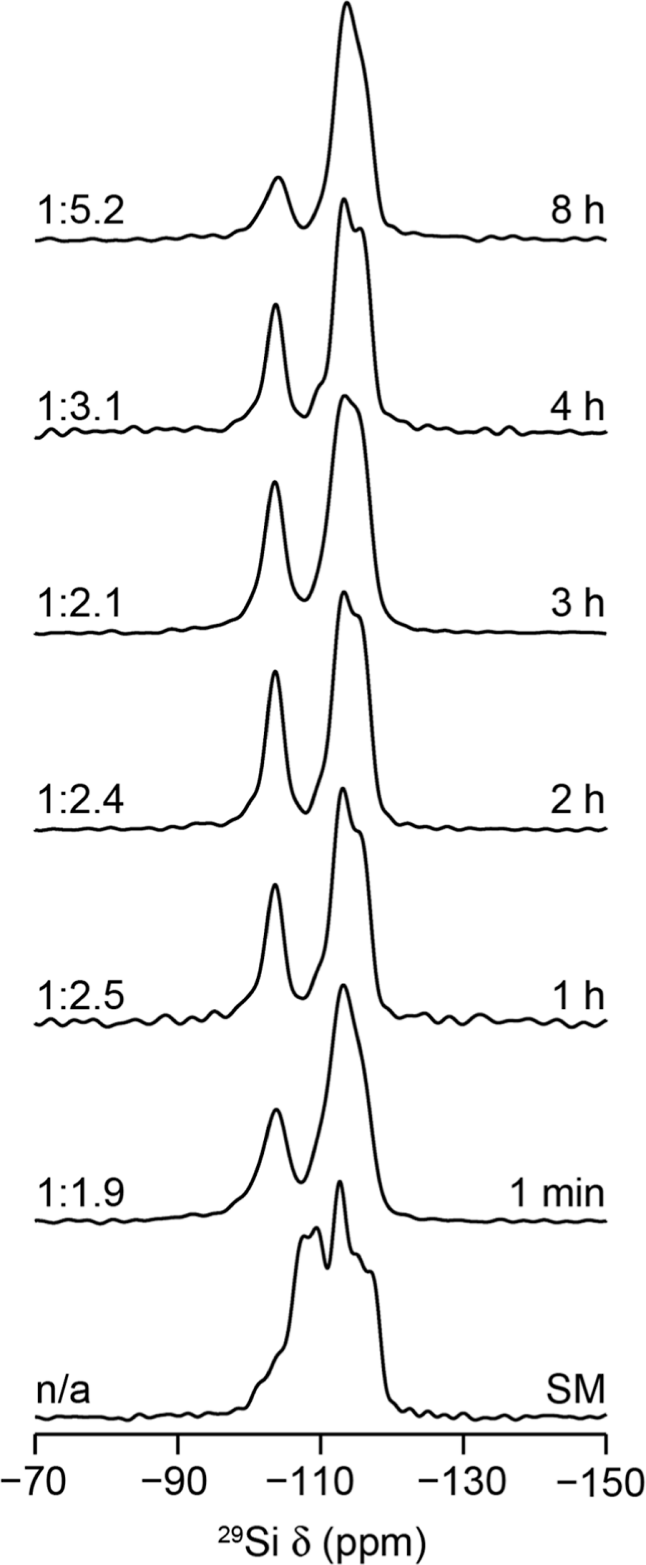
^29^Si (9.4 T, 14 kHz MAS) NMR spectra of products obtained from the hydrolysis of **UTL** in water at 92 °C using hydrolyses for varying times to study the induction period for any changes in the IPC-1P phase present. The Q^3^:Q^4^ (left) and reaction times (right) are indicated in each spectrum. Samples after 1 min and 8 h of reaction are expected to be IPC-2P* and IPC-2 (by diffraction), respectively, while those during the induction period after 1, 2, 3, and 4 h are all expected (from diffraction) to be IPC-1P. SM = starting material (UTL).

Table 2 shows that the isolated products of the reactions carried out at 100 °C possess the desired structural attributes (as measured by the interlayer spacing from *d*_200_) and, as such, the reaction is deemed to have proceeded successfully. Furthermore, the corresponding ^29^Si NMR spectra (see Figure 3) appear to follow the desired hydrolysis behavior: the parent **UTL** structure is changed after just 1 min of hydrolysis, exhibiting the expected Q^3^ and Q^4^ resonances. Figure 4 clearly shows how the proportion of Q^3^ and Q^4^ species changes over time, with the relative amount of Q^3^ increasing to a maximum at 1 h for IPC-1P, before decreasing again as rearrangement occurs to form IPC-2P at 4 h. This fits very well with the expected Q^3^:Q^4^ ratios calculated using idealized intermediates, as shown in Table 3.

**Table 3:** Comparison of Q^3^:Q^4^ Extracted from the ^29^Si MAS NMR Spectra in Figure 3 of Materials Obtained by Standard ADOR Hydrolyses of Ge-**UTL** in Water at 100 °C with the Corresponding Q^3^:Q^4^ Ratio That Would be Expected from Idealized Structural Models

hydrolysis time/min	idealized Q^3^:Q^4^	experimentally derived Q^3^:Q^4^
**UTL**	0	0
1	1:7.0	1:3.1
60	1:2.5	1:2.6
240	1:7.0	1:6.2
480	1:7.0	1:6.1

The results show that after 1 min, the Q^3^:Q^4^ ratio is different to that expected from the idealized version of IPC-2P* (1:3.1 versus 1:7). This is again because of the very fast changes occurring at this point in the reaction, making it difficult to sample the reaction at exactly the right point. After 1 h of reaction, the PXRD results suggest that major structural changes have stopped, and the ^29^Si NMR spectrum of this material shows a good agreement with the fully disassembled intermediate, IPC-1P. Diffraction then shows a reorganization to IPC-2P after the induction period. However, ^29^Si NMR spectroscopy suggests that the IPC-2P materials formed are more defective and silanol-rich than the ideal structural models would predict. This greater concentration of silanols may be caused by hydrolytic removal of any germanium that was found within the silica-rich layers of the **UTL** material, or through incomplete reintercalation of silicon species from the reaction solution, neither of which are modeled in the idealized structures. It could also arise from some Q^3^ defects that were present on the layers of the parent UTL material and remain unhealed throughout the process. Once again, however, we should say that the difference in measured Q^3^/Q^4^ ratios of, for example, 1.62 vs 1.7 (Table 3) is not significant enough for us to make concrete conclusions about extra defects based on these measurements.

While it is expected that the change in the structure of the ADOR hydrolysis products as the reaction progresses will result in changes to the ^29^Si NMR spectra in Figure 3, it is interesting to observe that changes to the local structure of materials are observed even when the Q^3^:Q^4^ ratios and the PXRD-derived *d*_200_ spacing suggest that structures are the same. For example, IPC-2P after 4 and 8 h of reaction have the same *d*_200_ spacing and the same Q^3^:Q^4^ ratios but slightly different lineshapes, indicating specific changes in the local structure that are not reflected in either the bulk PXRD or the overall Q^3^:Q^4^ ratio. Clearly, the local structural features are still evolving throughout the entire process.

#### 2.1.1 Induction Period

Figures 1 and 2 show that in the middle part of the reaction between disassembly of the **UTL** into IPC-1P and before any rearrangement into IPC-2P, there is a period where IPC-1P is the only intermediate formed despite the increase in reaction time. We call this the induction period. Lower reaction temperatures lengthen the lifetime of IPC-1P, with no further change to *d*_200_ observed at all for reactions carried out below 70 °C while reactions at 100 °C are too fast to sample sufficiently often.^20^ To balance the need for sampling with progress of the reaction, a series of standard hydrolysis at 92 °C in water is investigated here to explore whether there are changes to the local structure occurring during this induction period. The ^29^Si MAS NMR spectra of the products are shown in Figure 4.

Looking at Figure 4, the Q^3^:Q^4^ ratios for the isolated products are as expected for ADOR hydrolyses taking place under these conditions, with the dominant product in all reactions >1 h being IPC-1P, with the exception of the 8 h experiment (where Figure 2 confirms that the IPC-2 product would be expected). Figure 2 also shows that the materials present after 1, 2, 3, and 4 h (i.e., during the induction period) exhibit very similar PXRD patterns. It is clear, however, that during this time, the local structure of the intermediate materials constantly changes, as evidenced by the differences in the ^29^Si spectral line shape and the variation in the proportion of both Q^3^ and Q^4^ environments in the structures.

The disordered nature of the IPC-1P intermediate makes it difficult to identify exactly which structural and environmental changes are observed when comparing the spectra in Figure 4, yet a few general conclusions can be drawn. When comparing the Q^3^ resonances for materials at 1 min with IPC-1P at times >1 h, the sharper line shape seen for the latter makes it clear that the silanol distribution is more ordered. This narrower line shape persists again until the material changes phase by reorganization to IPC-2P. More ordered silanols may be expected for IPC-1P materials as they are expected to occur only at the interface of the interlayer region of the silicon-rich 2D sheets. However, the spectra in Figure 4 also indicate that there is a constant change in the local structure of the Q^4^ Si environments in the siliceous IPC-1P structure, prior to any reintercalation of aqueous silicate species occurring, which would be clearly identifiable by a change in the *d*_200_ spacing in the PXRD. Exactly what structural rearrangements happen during this period is unclear as the disordered nature of the IPC-1P structure and the large number of crystallographically distinct silicon sites in the material make it difficult to identify exactly what the different contributions to the Q^4^ line shape are. X-ray PDF studies^35,36^ strongly suggest that the disassembly process is multistep, with fast loss of Ge from the d4r units that link the silica rich layers followed by a slower loss of silicon from these d4r units. Ge-**UTL** is striking for its tendency to form Ge s4r, or 4r “faces” of Ge_4_O_4_ rings within the d4r, which makes the “interlayer region” increasingly hydrolytically unstable and therefore ideal for ADOR-type transformations.^37^ It is feasible to suggest that the formation of IPC-1P proceeds through an intermediate that has vestiges of silicon-rich s4r units that are likely present in the parent **UTL** material. The initial stages of disassembly therefore proceed by fast loss of some, or most, of the germanium in the material to form IPC-2P* (after 1 min), which leaves the layers connected by the remaining silicon species in the interlayer space. These are then lost more slowly to form IPC-1P. During the induction period, the local structure of IPC-1P subtly changes until reintercalation/reorganization of silicon becomes favorable and IPC-2P can eventually be formed. The similarity of the spectral lineshapes for the 1 and 4 h samples in Figure 4 is therefore not so surprising. The changes observed between these time points at 2 and 3 h must be part of the subtle reorganization process (although a process that does not significantly affect the overall proportion of Q^3^ and Q^4^ species, but presumably just their distribution). Whatever the specific changes to the local structure that occur, it is clear that reorganization of IPC-1P is necessary in order to allow for the silicon reintercalation during the organization step of the ADOR process.

There is a known preference for germanium to locate at the T6 position in Ge-**UTL**, which lies on the surface of the silica-rich layer and is connected through T–O–T linkage to the d4r.^26^ This position should not actively take part in the ADOR process and is not taken into account in the idealized structure calculations. However, germanium in this “silicate layer” position will still be hydrolyzed upon contact with water. This may offer an explanation as to why the Q^3^:Q^4^ ratio observed is higher than that expected for IPC-1P as one germanium removed here will produce four Q^3^ silanol groups that are not predicted from the idealized structures. Any formation of defects in this position may have to be healed before silicon could be fully reintercalated into the structure. However, the differences between ratios of 1.6 and 1.7 are probably not significant enough to make strong conclusions about the defects.

### 2.2 Following the ADOR Process Using ^17^O NMR Spectroscopy

Previous sections highlighted the importance of XRD and ^29^Si NMR spectroscopy techniques to study the ADOR process. The former underlined the effect of ADOR transformations on the long-range order of the materials and intermediates formed and that through adaption of the hydrolysis conditions the products of ADOR transformations of **UTL** can be controlled. The latter showed how the local structural disorder of these ADORable intermediates is also influenced by hydrolysis conditions and in some cases how this can paint a picture that is complementary to that observed by XRD.

Introduction of the ^17^O isotope to the reaction, and its subsequent study using ^17^O NMR spectroscopy, provides further insight into the mechanism of the ADOR process by shining a light on specific zeolite reactivity. Employing ^17^O-enriched materials as reagents in ADOR reactions enables the possibility of studying specific interactions at particular points in the reaction pathway and can provide information about how reversible bond lability and irreversible bond hydrolysis processes are facilitated in ADOR transformations. The high costs associated with the use of ^17^O-enriched reagents mean their use in an extensive suite of experiments to explore the effect of the ADOR process on the oxygen local environment in **UTL** materials is not feasible. Instead, using the knowledge of ADOR process tunability and control, as explored above, it is possible to design a set of specific experiments that target specific materials generated in the ADOR process for further study. This, in turn, allows for more efficient extraction of information from interesting structures, revealing information about the ADOR mechanism and the roles of different intermediates.

The results in this section follow on from previously published work that aimed to uncover the mechanism of the ADOR process under low volume (high solid/water) conditions.^22^ Low volume conditions are different from those studied in the current work because under low volume conditions IPC-1P is disfavored and only IPC-2P is formed. Additional to the new mechanism for hydrolysis in low-volume conditions, the previous study by Bignami et al.^22^ revealed that the rearrangement and exchange of framework oxygen species with those in the hydrolyzing solution was more extensive than first thought. Significant exchange of ^17^O into framework sites in the two-dimensional zeolite layers was observed using ^17^O MQMAS and ^29^Si/^17^O HETCOR experiments at high magnetic fields.^38^ This result confirmed not only the expected presence of Si–^17^OH silanols formed irreversibly through hydrolysis but also the formation of Si–^17^O–Si linkages in the layers of hydrolyzed IPC-2P intermediates.

By completing hydrolysis reactions in the presence of H_2_^17^O, this section uncovers more about the rate at which reversible bond lability and irreversible bond hydrolysis processes occur in Ge-**UTL** and its related IPC-2P intermediate structure.

**Figure 5:**
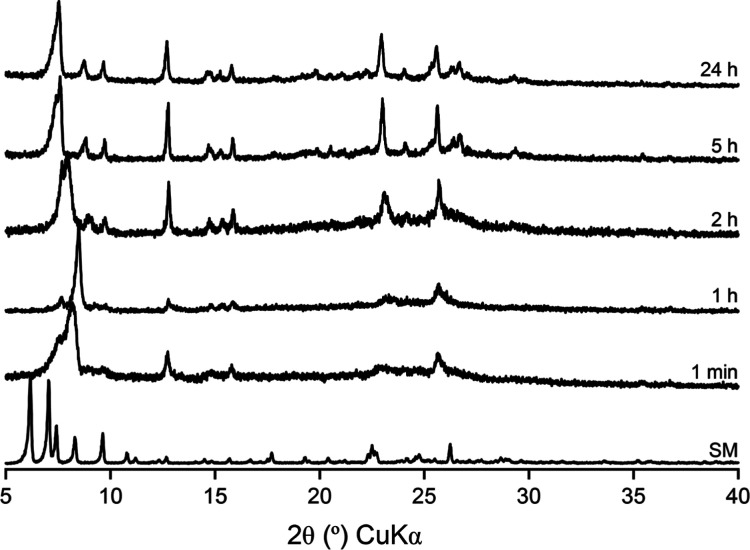
PXRD patterns of products obtained from the continual hydrolysis of Ge-UTL in water (3.5% H_2_^17^O) at 92 °C. The hydrolysis time is indicated on each trace. SM is the starting material **UTL**.

**Figure 6:**
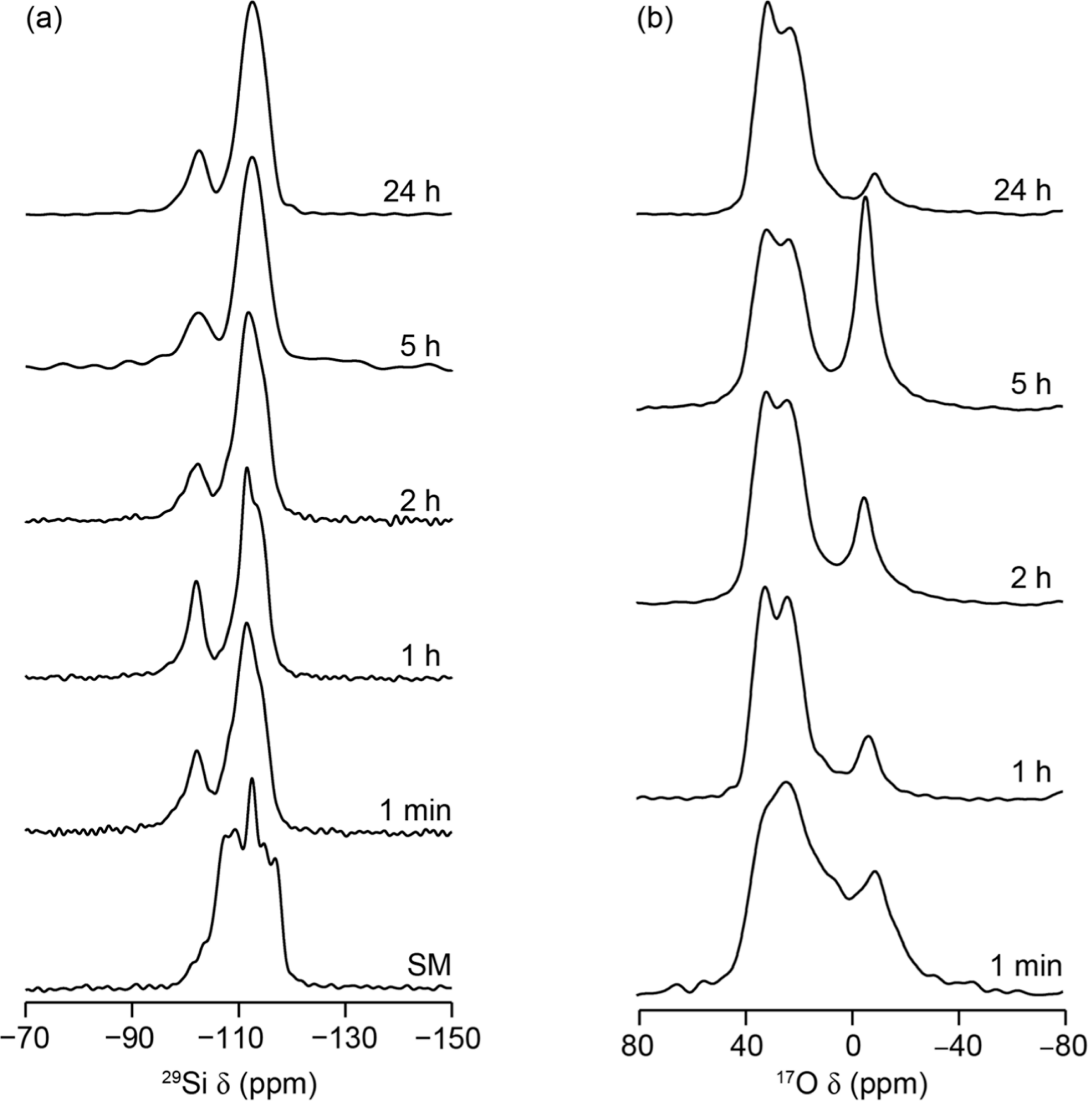
(a) ^29^Si (9.4 T, 14 kHz MAS) quantitative and (b) ^17^O (20.0 T, 14 kHz MAS) short flip angle NMR spectra of products obtained from the continual hydrolysis of Ge-**UTL** in water with a 3.5% concentration of H_2_^17^O at 92 °C. Hydrolysis time is indicated on each trace. SM is the starting material **UTL**.

Hydrolyzing Ge-**UTL** with a solid/water of 1:80 successfully resulted in the high-volume ADOR hydrolysis mechanistic pathway, as shown in Figure 2. This ratio, combined with 3.5% H_2_^17^O, provided an adequate amount of ^17^O incorporated into disassembled materials to facilitate an NMR spectroscopy study on a feasible time scale. The PXRD patterns and quantitative (i.e., short flip angle) ^17^O and ^29^Si NMR spectra of the isolated products of this reaction are displayed in Figures 5 and 6, respectively.

The diffraction patterns in Figure 5 confirm that the expected hydrolysis pathway is largely followed and is in qualitative agreement with the observed Q^3^:Q^4^ ratio derived from the ^29^Si MAS NMR spectra in Figure 6. In this instance, the induction period is shorter than typically expected for a 92 °C reaction under “high-volume” conditions. This is likely caused by the higher solid/water ratio of 1:80 (previous studies used 1:200). This is a common observation when higher solid/water ratios are used.^22^

**Figure 7:**
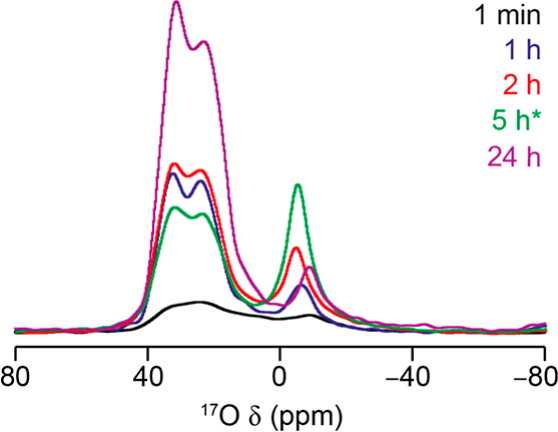
^17^O (20.0 T, 14 kHz MAS) short flip angle NMR spectra of products obtained from the continual hydrolysis of Ge-**UTL** in water with 3.5% H_2_^17^O at 92 °C. Spectra are scaled to take account of both the number of transients averaged and the mass of sample in the rotor. Hydrolysis time is indicated on each trace. The small amount of the 5 h sample recovered (denoted * on the figure) was studied by packing this into a 4 mm PTFE HRMAS NMR insert which was then packed inside the rotor.

Alongside the hydrolysis taking place in the abovementioned reaction, the ^17^O MAS NMR spectra acquired at high field provide evidence of additional processes taking place during ADOR hydrolysis that confirm the exchange of **UTL** framework oxygen species. Both the nature of this enrichment and the rate at which it occurs is surprising; the framework ^17^O NMR signals can be seen in these materials even within 1 min of reaction in enriched water, which is comparable to the rate at which the rapid ADOR hydrolysis occurs under these conditions. To further understand the surprisingly rapid enrichment process observed, the signal intensities of the short-flip ^17^O MAS NMR spectra are compared in Figure 7.

From Figure 7, it is clear that rapid framework enrichment takes place at short hydrolysis times, with a high rate of framework exchange up until IPC-1P becoming the dominant phase at 1–2 h. As the reaction time increases and the material eventually transforms to IPC-2P, exchange of the framework oxygens continues, producing a highly enriched material after 24 h. The lower absolute level of enrichment seen for the material at 5 h reaction time likely arises from the low volume of sample recovered from this reaction. This had to be packed with a 4 mm PTFE HRMAS insert within the rotor. Although this, in principle, can be accounted for in terms of the mass, it is not clear if the measurement is strictly comparable. Furthermore, this sample also has a surprisingly high level of water present (signal at 0 ppm), which will lead to differences after scaling for the sample mass.

**Figure 8:**
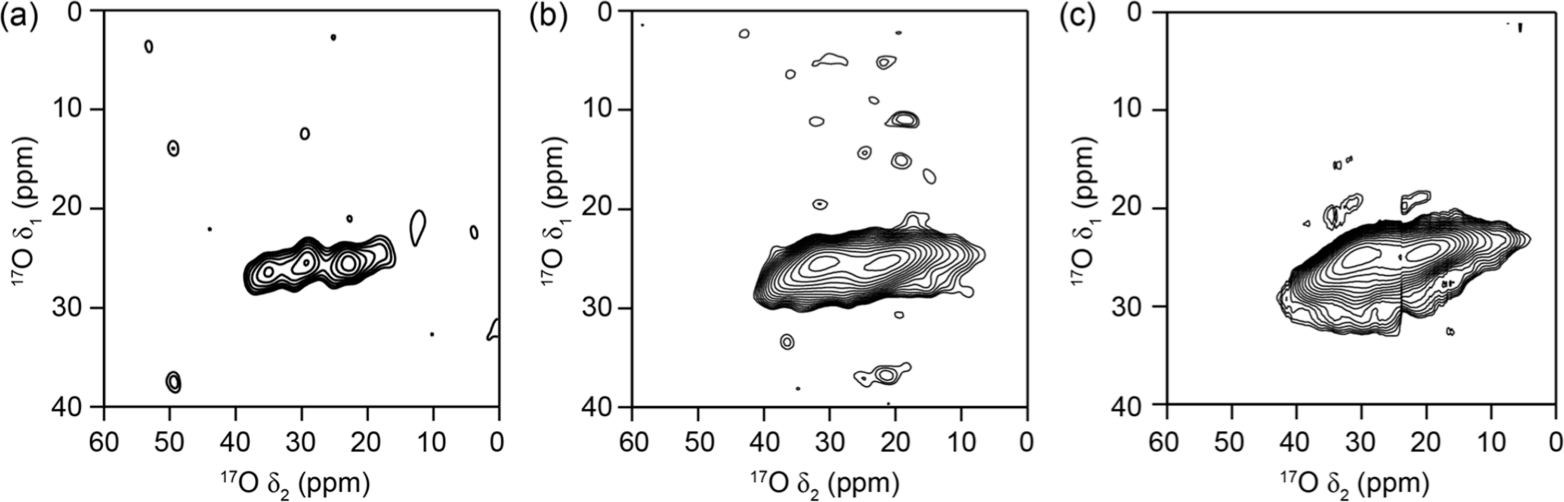
^17^O (20.0 T, 14 kHz MAS) and proton-decoupled ^17^O MQMAS NMR spectra of products obtained from the continual hydrolysis of Ge-**UTL** in water with 3.5% H_2_^17^O at 92 °C. Spectra displayed correspond to samples hydrolyzed for (a) 1 min, (b) 2 h, and (c) 24 h.

To aid spectral deconvolution of the complex lineshapes in the ^17^O MAS NMR spectra in Figure 7 and learn more about the local structural environment of the newly exchanged oxygen species in the materials, two-dimensional high-resolution solid-state NMR experiments were performed. Proton-decoupled ^17^O MQMAS NMR experiments for selected intermediates are shown in Figure 8.

The spectra in Figure 8 show that as hydrolysis time increases, there is a qualitative increase in sensitivity of each MQMAS NMR spectrum (measured simply by the signal-to-noise ratios of ∼1:3.1:5.4), which have been recorded using the same spectral parameters and similar sample masses. With the exception of Figure 8a, where a small amount of germanium is still likely to be present, the materials in each spectrum are purely siliceous, taking the form of layered Q^4^ tetrahedral silicate sheets terminated by Q^3^ silanol groups. Therefore, potentially resolvable resonances in these spectra would correspond to oxygen present in framework Si–^17^O–Si linkages and Si–^17^OH silanols.^22^ Using published examples from the literature,^39,40^ the resonance observed can be best assigned to Si–O–Si linkages within the structure of the intermediates. The lack of Si–^17^O–Ge signals (seen in previous work using mechanochemistry^33^) confirm that the Ge is removed quickly from the framework during hydrolysis. The crystal structure of the **UTL** framework contains 23 crystallographically distinct oxygen sites, 21 of which are found in the siliceous layers. However, the complexity of the **UTL** framework and the broadening resulting from distributions of chemical shift and quadrupolar parameters from the disorder present in these phases precludes the resolution and assignment of specific crystallographic sites to regions within the observed resonance.^41^

Interestingly, there is no evidence for signals from isotopically enriched silanols in the ^17^O MQMAS spectra (despite clear evidence for silanol formation in ^29^Si NMR spectra). Considering their role in the hydrolysis reaction and their interactions with aqueous species in the system, possible reasons for this could include a back reaction with water in the air (although this is unlikely given the sample handling) or more likely rapid relaxation arising from proximity to, or exchange with, water in the pores. This was observed in previous work on ADOR layered intermediates produced in low-volume reactions, where the Si–^17^OH signal was not seen in MQMAS experiments unless very strong ^1^H decoupling was used, and the ^1^H–^17^O CP signal was extremely poor unless at very low temperature.^22^^1^H–^17^O CP MAS NMR experiments at room temperature were also unsuccessful at room temperature for the samples studied here, leading to the conclusion that the rapid relaxation and/or ongoing exchange with the water of the terminal silanol species is occurring in these samples. Although these signals should be present in the ^17^O MAS spectra, previous work on similar ADOR intermediates showed that this signal was overlapped with the broader Si–^17^O–Si signal and (especially given the complexity of the UTL structure) difficult to quantify for these samples.

### 2.3 Incipient Wetness Impregnation of IPC-2P

**Figure 9:**
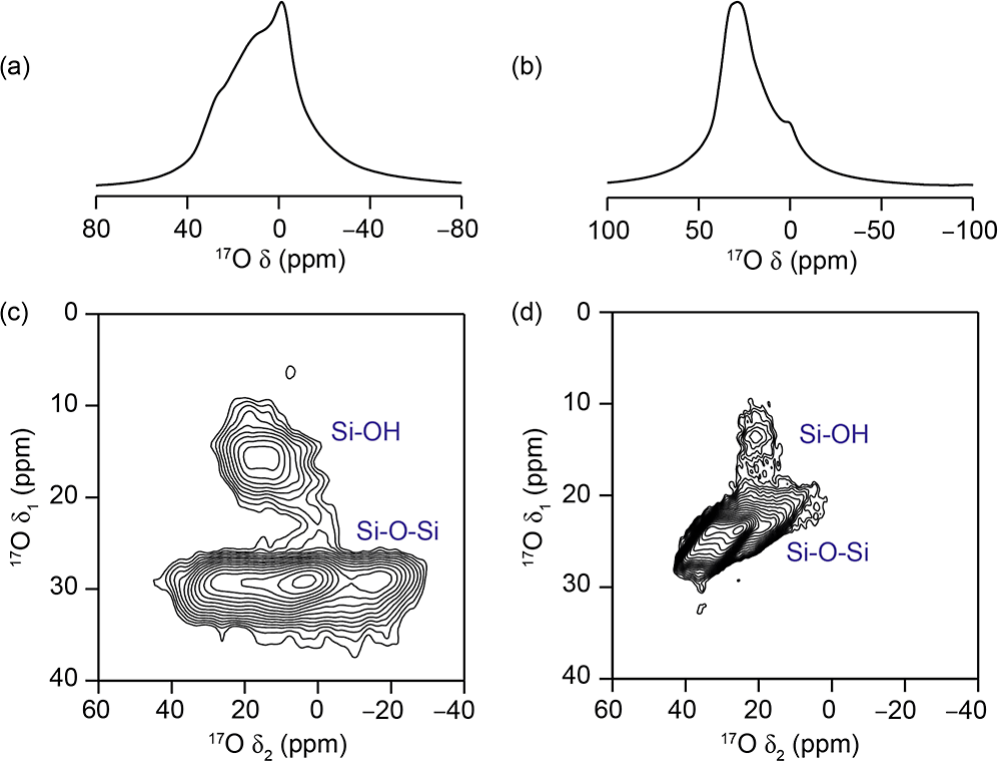
(a,c) (14.1 T, 14 kHz MAS) and (b, d) (23.5 T, 20 kHz MAS) (a,b) ^17^O quantitative short flip angle and (c,d) proton-decoupled ^17^O MQMAS NMR spectra of IPC-2P postsynthetically enriched in ^17^O by incipient wetness impregnation using H_2_^17^O(l).

Recent results have demonstrated the rapid but reversible lability of Si–O–Si bonds in aluminosilicate zeolite frameworks even at room temperature in the presence of water.^18,19^ This indicates that in addition to the ADOR processes that alter the structure of the intermediates, there may be other dynamic processes occurring that do not change the overall structure of the material but could lead to isotopic exchange in the reactions studied here. It is desirable to distinguish whether the exchange of framework oxygens observed in previous ADOR hydrolyses and NMR studies is a consequence of the ADOR processes itself or whether this exchange occurs independently from hydrolytic disassembly and rearrangement. To test whether this was the case, we added ^17^O-enriched water dropwise to a sample of IPC-2P over a very short period of time (i.e., using an incipient wetness approach). The IPC-2P material was dried before the introduction of H_2_^17^O(l) by direct dropping of ∼100 μL of 40% enriched water onto ∼160 mg of sample over a period of about 10 min before the sample was then allowed to dry in air. ^17^O NMR spectra of the postsynthetically treated ^17^O-enriched material were then collected at two different fields, as shown in Figure 9.

From Figure 9, it is clear that simple incipient wetness also facilitates the isotopic exchange of oxygen atoms in the framework with those from the water, with evidence of both Si–^17^O–Si and Si–^17^OH resolvable in MQMAS spectra recorded at both fields (14.1 and 23.5 T). At 14.1 T, signals are seen at δ_1_ ≈ 26–35 ppm (Si–^17^O–Si) and δ_1_ ≈ 16–23 ppm (Si–^17^OH).

The evidence for framework enrichment observed in the incipient wetness experiments helps to provide a better understanding of the bond lability processes occurring in ADOR intermediates. First, the extent of the enrichment observed here is significant, confirming that this exchange is rapid, even at room temperature and with relatively little water. The mechanism behind this enrichment therefore must not require a carrier phase, acidic conditions, heat, or a large excess of water molecules to facilitate it. Second, as no ADOR processes are occurring in this sample (IPC-2P is a Ge-free intermediate phase and not expected to be hydrolytically vulnerable) or are expected on this time scale, the enrichment exchange is likely independent of any ADOR-reactive species. Both Si–^17^O–Si and Si–^17^OH linkages are enriched, which indicates an extremely dynamic system even under these very mild conditions. Furthermore, the extent to which the material is enriched overall points to the fact that a greater proportion of the silicate linkages than those involved in the ADOR surface reintercalation processes have been exchanged, meaning that those linkages within the two-dimensional silicate sheets are also reactive under these conditions. The fact that resonances due to both the Si–^17^O–Si and Si–^17^OH groups are visible indicates that the lack of Si–^17^OH signals in Figure 8 is likely due to fast exchange with the excess water that is clearly present in these samples (Figure 7), but which is not present when wetness impregnation is used.

While the spectra in Figure 9 confirm room-temperature framework lability in the IPC-2P intermediate by simple hydration with small amounts of water, several questions about the mechanism for this process remain. The IPC-2P material, although not compromised in the process, is inherently defective, containing many Q^3^ silanol defects. The role that these play in the structure and reactivity of the material is not fully understood. To further understand the processes occurring that lead to the surprising framework oxygen exchange observed in Ge-**UTL** and its ADOR-derived daughter material, further experiments exploring alternative methods for framework enrichment and following this as a function of time are desired.

## 3 Conclusions

The work presented here has provided greater insight into the mechanism of the ADOR process and the changes in local structure that drive this. NMR has been used for the first time to systematically study the changes that occur during the induction period seen in many ADOR reactions. Although the exact changes in local structure are difficult to determine from the complex spectral lineshapes seen, it is clear that these changes are occurring throughout this period despite the lack of any change in the PXRD patterns over this time. The overall mechanism of the ADOR process is therefore very complex with competing processes (significant structural changes shown in Figure 1 as well as local and reversible changes) occurring at different rates, which are extremely dependent on the conditions used. In addition to the more significant structural changes that define the ADOR process itself (Figure 1), the work described here also demonstrates the remarkable lability of the Si–O–Si bonds in the bulk layers of the intermediate materials using ^17^O NMR spectroscopy. Not only are these seen to be reactive under the conditions of the ADOR reaction itself, surprisingly so given the clear retention of these layers by PXRD, simple incipient wetness experiments have shown this lability exists on exposure to water at room temperature, with extensive enrichment of the Si–O–Si bonds in the zeolitic layers. This reactivity is remarkable given the very low volume of water used, the lack of any hydrolytically sensitive X–O–Ge bonds, and the absence of the Brønsted acidic Si–O–Al linkages present in the aluminosilicates studied previously. It is clear that a range of processes and taking place different time scales in an ADOR reaction, with rates that can differ significantly with changes to the conditions used. This emphasizes the complexity of this process, but the huge potential to control the reaction and the intermediates formed if greater mechanistic and atomic-scale insight could be obtained.

## 4 Materials and Methods

### 4.1 Zeolite UTL Synthesis

GeO2 (15.68 g, 150 mmol) was added to a solution of (6*R*,10*S*)-6,10-dimethyl-5-azoniaspiro[4.5]decane hydroxide (DMAD–OH, 0.625 M, 240 cm^3^, 150 mmol) and mechanically stirred for 15 min, before SiO2 (Cab-O-Sil M5) (17.98 g, 300 mmol) was added portionwise under stirring. The mixture was stirred under high shear for 30 min, forming a reaction gel of composition
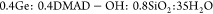


The gel was sealed in Teflon-lined steel autoclaves and heated to 180 °C for 7 days, before quenching, cooling, and filtering the product. The product was washed with distilled water and acetone and dried at 80 °C. Calcination was performed at 575 °C for 6 h. Product Si/Ge = 4.5, as measured by energy-dispersive X-ray analysis. Further details of characterization (XRD, SEM, and ^1^H and ^29^Si NMR) can be found in the Supporting Information (Figures S1 and S2).

### 4.2 ADOR Process

A typical ADOR procedure is as follows. Zeolite **UTL** Si/Ge = 4.5 (600 mg) was hydrolyzed in distilled water (120 cm^3^) (solid/water 1:200) at 92 °C with stirring at 400 rpm for 8 h. Samples were taken from the reaction at specified time intervals (for 1–5 min; every minute, for 5–60 min; every 5 min and for 60–480 min; every 30 min) in the minimum amount of hydrolyzing solution. Isolated intermediates were filtered, washed with distilled water, and dried at 80 °C for 10 min, before PXRD analysis.

### 4.3 ADOR Process Using ^17^O Enriched Water

Calcined zeolite **UTL** (Si/Ge = 4.5) (800 mg) is combined with a 3.5% solution of H_2_^17^O (prepared from distilled water and 20% H_2_^17^O enriched water) (64 mL). The mixture is heated to 92 ^*◦*^C for 24 h under rotation at 400 rpm. Samples are taken from the reaction at 1 min and 1, 2, 5, and 24 h such that the solid/water ratio in the flask remains approximately constant. The solids are filtered and washed with the minimum amount (<5 mL) of distilled water and then dried at 80 ^*◦*^C for 10 min. Solid/water = 1:80 (by mass).

### 4.4 Incipient Wetness ^17^O Enrichment of IPC-2P

Isolated IPC-2P (100 mg) is impregnated dropwise with 40% H_2_^17^O (100 μL). The rate of addition was relatively slow to ensure that the water could be adsorbed by the material. In practice, the addition of enriched water took ≈10 min at room temperature.

### 4.5 NMR Spectroscopy

Solid-state NMR experiments were performed using Bruker Avance III spectrometers equipped with wide-bore magnets operating at magnetic field strengths, *B*_0_, of 9.4 (for ^29^Si) and 14.1 T, 20.0 or 23.5 T (for ^17^O). Powdered samples were packed into 4 or 3.2 mm ZrO_2_ rotors and rotated at rates between 10 and 20 kHz. Chemical shifts are quoted in ppm relative to **Si** (CH_3_)_4_ or H_2_^1**7**^**O** (l), measured in the first case using secondary (solid) references of octakis(trimethylsiloxy)silsesquioxane (Q8M8, OSi(CH_3_)_3_ δ_iso_ = 11.3 ppm). Typical recycling intervals were 120–140 s (^29^Si) and 1 s (^17^O). ^17^O MQMAS experiments were acquired using a triple-quantum amplitude modulated z-filter pulse sequence with a final central-transition selective 90° pulse. Spectra are shown after a shearing transformation and referenced using the convention described in ref (42).

## Supporting Information Available

Further information on the characterization of the starting UTL material (XRD, SEM, and ^1^H and ^29^Si NMR) (PDF)
The research data underpinning this publication can be accessed at 10.17630/b72e4b38-bfc5-4b72-b1aa-533f8fa250f2
