# Supplementary material for: Investigations of the ADOR Process Using Solid-State NMR Spectroscopy
Source: Cryst Growth Des. 2023 Nov 15;23(12):8991–9000. doi: 10.1021/acs.cgd.3c01037 (PMC10704408; doi:10.1021/acs.cgd.3c01037)
Supplement: Supplementary file 1 — cg3c01037_si_001.pdf [file cg3c01037_si_001.pdf]

Investigations of the ADOR process using solid-state NMR spectroscopy

Cameron M. Rice, Olivia Dovernor, Russell E, Morris and Sharon E Ashbrook

School of Chemistry, EaStCHEM and Centre of Magnetic Resonance, University of St Andrews, Purdie Building, St Andrews KY16 9ST UK

Dedicated to Professor Svetlana Mintova

SUPPLEMENTARY INFORMATION

S1 Characterisation of UTL

UTL was synthesised according to Materials and Methods Section in the main paper producing a highly crystalline material, stable to calcination at 550 °C SEM and EDX analyses show a single phase of large platelet type crystals, typical for UTL materials, with a Si/Ge ratio of ≈ 4.5

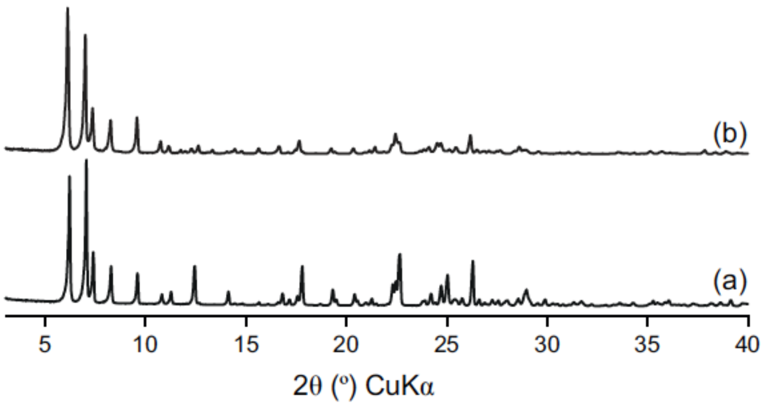

Figure S1: Powder X-ray diffraction patterns for (a) reference UTL material and (b) synthesised Ge-UTL

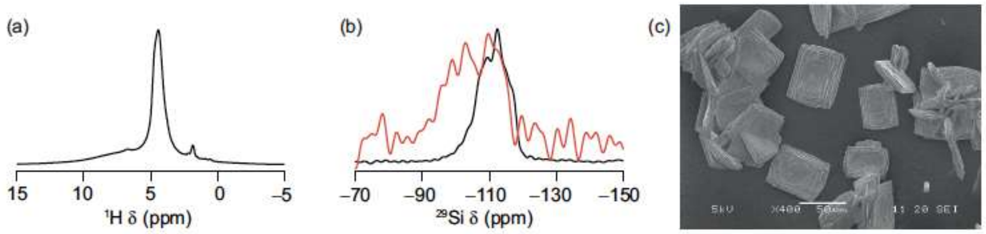

Figure S2 (a) ^{1}H and (b) ^{29}Si (14 kHz MAS, 9.4 T) NMR spectra and (c) SEM image of synthesised GeUTL. Red overlay in (b) corresponds to 1H^{29}Si CP spectrum (5000 μs contact time)
